# Avoiding global deforestation by taxing land in agricultural production: the implications for global markets

**DOI:** 10.1186/s13021-025-00291-7

**Published:** 2025-03-13

**Authors:** Eric C. Davis, Maros Ivanic, Brent Sohngen

**Affiliations:** 1 United States Department of Agriculture-Economic Research Service, Kansas City, MO 64105 USA; 2 Department of Agricultural, Environmental, & Development Economics, The Ohio State University, Columbus, OH 43210 USA

## Abstract

The projected growth in population and incomes is expected to create pressure to convert forestland into farmland. At the same time, the increasingly negative climate impacts are expected to generate further pressure to enhance the terrestrial carbon sink. Even though these goals are incompatible as reversing the deforestation trend by afforesting cropland would result in negative market impacts such as higher food prices, using the GTAP and GTM models, we find that these impacts would be relatively small if the goal of preserving 144.2 million hectares of forestland that otherwise would be converted to agricultural land by 2033 is achieved through a tax on land use in agricultural production. As to the economic price for doing so, the avoided deforestation would in most regions of the world result in less agricultural output and higher market prices. This is estimated to impact the well-being of global consumers by $119.7 billion, which translates to a global average cost of $13.78 per person in 2033.

## Introduction

In 2020, there were 4.1 billion hectares of forestland globally covering roughly 31.4% of Earth's landmass [1]. These forests play an important role in maintaining and growing the land-based carbon sink by sequestering about 30% of GHG emissions currently [2]. This is because of forests proven ability to sequester carbon not only in the stems of trees [3] but in their soils as well [4]. With increasingly changing climate dynamics, the pressure to enhance the terrestrial sink is growing, and one of the tools to achieve this is afforestation. As such, there is growing attention being placed globally to afforestation efforts, which is highlighted by the UN’s declaration of the years from 2021 through 2030 as the UN Decade of Ecosystem Restoration [5]. Many of the Nationally Determined Contributions (NDCs) countries have submitted, which detail their plans to keep warming under the 2C threshold, also rely to a significant degree on afforestation projects [2].

The global gains that can be realized from afforestation, however, are uncertain, with broad estimates of cumulative sequestration ranging from 42 gigatonnes to 700 gigatonnes by the end of the century [6–10]. In addition, the amount of land conversion that would be required also varies greatly, with estimates ranging from 0.3 billion hectares to 2.8 billion hectares [6–9]. In many cases, these results suggest massive losses in the amount of pasture and cropland that would be available. This puts into question the feasibility of attaining these results, as almost 90 percent of deforestation worldwide is undertaken to expand the amount of agricultural land [11]. There are many reasons for this. For example, as nations develop and disposable incomes rise, consumers often increase meat consumption. Population growth is also a driver. Currently, the United Nations’ medium variant population projections suggest that between 2023 and 2033 the worlds’ population will grow by 8.8 percent, a gain of 707.1 million people [12]. And where population growth is especially high, which is the case at present throughout Africa (2023 to 2033 population growth: 25.0%: 360.6 million), there is likely to be greater conversion of forestland into farmland. Still, some research has suggested that substantive gains in the carbon sink (+ 23.8 PgCo2e/yr) can be made without sacrificing agricultural land [13].

Carbon benefits from afforestation are heterogenous. Therefore it is important to understand the potential impact across regions. For example, afforestation in boreal zones provides the smallest net benefits, as the carbon sequestration benefits of conifer forests are mitigated by the reduction in albedo in winter [14, 15]. Coniferous forests have also been shown to increase soil organic carbon less than broadleaf forests [4]. Conversely, tropical forests have been linked with a strong positive result from afforestation, as the carbon sequestration benefit is complemented by a cooling impact due to changes in both albedo and evapotranspiration, and with temperate forests, the impact of afforestation is also shown to be positive, albeit less strongly so due to a winter warming effect [14].

South America and Sub-Saharan Africa are thus two regions that show much potential for afforestation. It is estimated that these two regions hold at least 50% of the potential for global gains while regions such as Northern Africa and the Middle East show little promise as forest growth rates are quite low [8]. Thus, it is promising that the African Forest Landscape Restoration Initiative (AFR100), which aims to afforest 100 million hectares by 2030, has roughly 70% of its commitments coming from Sub-Saharan nations [16]. There are other nations that have a big potential for gains from afforestation, but they face challenges. India, for example, has a great potential to increase its carbon sequestration through afforestation, but its massive population and food security worries limit its ability to consider such actions. The United Nations suggests that India’s population will grow less rapidly than the world average between 2023 and 2033 (+ 8.5%), but due to its sizable base, the increase is large nonetheless: 120.5 million. India’s NDC does not entertain the idea of agricultural land being re-purposed and instead primarily considers targeting land that may not be well suited for afforestation [17]. Moreover, as land-use decisions in one country also tend to have impacts that spillover into many others [18, 19], analysis needs to be conducted at a global scale.

Several notable modeling attempts have been made in this direction. Steinbuks and Hertel [20] developed their global partial equilibrium FABLE model (Forestry, Agriculture, Biofuels, Land Use, and Environment) to identify the optimal land use choices given the competing demands of meeting greenhouse gas targets and satisfying demand for food, bioenergy, forest products, and ecosystem services. While FABLE is a powerful tool for examining the optimal trajectory of various land uses under specific demand assumptions, it is focused on specific sectors of the economy and neglects general equilibrium and household welfare effects [20, 21]. Other efforts have used the Global Trade Analysis Project (GTAP) computable general equilibrium model to address such questions, but its standard model does not well account for land-use changes. This prompted the creation of the Land Use and Land Cover Database within the GTAP framework, which incorporates forestry remote-sensing products [22]. The GTAP Agro-Ecological Zone (GTAP-AEZ) model further strengthened the GTAP model’s ability to analyze land use changes, with the use of spatially explicit global land use data and through the incorporation of intra- and inter-regional land and land-based greenhouse emissions heterogeneity [23]. While the GTAP-AEZ model benefits from the inclusion of competition among different crops, grazing, and forest-based uses, it has limitations, primarily due to its irregular updates. The KLUM@GTAP framework integrated the Kleines Land Use Model (KLUM) with an extended yet static version of GTAP called GTAP-EFL to evaluate the impact of climate change on cropland allocation. KLUM is a global agricultural land-use model that connects the economy to global crop allocation to maximize producer returns under specific risk assumptions, and GTAP-EFL separates energy factors from intermediate inputs and incorporates them into capital, while also considering CO2 emissions. KLUM@GTAP substitutes the land allocation mechanism within GTAP-EFL by utilizing regionally aggregated area changes in cropland determined by KLUM to update cropland shares in GTAP-EFL [24].

More recent modeling attempts of land-use change have begun to move away from comparative static analysis though. For example, the comparative-static GTAP model, supported by the GTAP-AEZ database, considers land market effects, which were identified as significant in driving results by Stevenson et al. [25]. This model has been utilized to evaluate the impact of crop intensification on land use. It, however, has been critiqued, as global aggregates may mask localized shifts that can have implications for ecologically significant areas [26]. In addition, in the standard GTAP model, from the modeling standpoint, it is difficult to model a sector individually, e.g., by using a different assumption on its production function, as this requires a lot of additional code to assure the integration of all sectors. There are 65 market sectors covered in the latest version of the GTAP database. The GTAP model’s general equilibrium nature means that all these markets are cleared with market-clearing prices. As the model applies nested CES production functions to all sectors and differentiates them by parameters only, to model one sector, in this case forestry, differently from the rest would require a major redesign of the model. While not impossible, this project will attempt to get around these concerns and assess the impact of various likely land-use scenarios by melding the standard comparative-static GTAP model with the Global Timber Model (GTM), which is a dynamic optimization model that has been specifically designed to analyze the relationship among land rents, forested land cover, and carbon sequestration through forests and thus is better able to examine the impact of afforestation or avoided deforestation on the carbon sink [27]. The latest versions of both models were used. For the GTAP model, this meant using data which has 2017 as its base year. This data was then updated to the base year (2023) using data from the Centre for Prospective Studies and International Information (CEPII) [28] to account for the projected changes in gross domestic product (GDP), population, and productivity for each country in the world. Similar steps were taken to update and recalibrate the GTM model. As most forest inventory data are not updated annually, the GTM model updates data for regions with inventory information approximately every five years, and it updates data for other regions using the Global Forest Resource Assessment [29].

## Connecting the GTAP and GTM models

In our work, we use the current version of the GTM model. 1We also use the standard GTAP model version 6.2. 2Both the GTAP and GTM models represent widely used models for policy analysis. In the case of GTAP, the model is used to describe the implications of various policy instruments for national and global markets, including prices, output and trade. The GTM model is, on the other hand, capable to translating the impacts of population and global growth, and the changes in land returns, to changes in global forest stocks and calculate the impacts on total carbon contained in forests. Because the regional amount of accessible land is fixed, we use the changes in forest coverage in the GTM model to calculate the change in agricultural land, treated as the residual land.

Even though the GTAP and GTM models capture important aspects of the global economy and the biophysical world, they do not model certain economic responses, and, instead, consider several important variables exogenous. In the case of GTAP, the model assumes that the amount of agricultural land is fixed, while the GTM considers the agricultural returns to land fixed. By combining the two models, we can fill each model’s missing behavior for a more complete description of the global impacts.

The combination of the two models means that we use the GTAP model to create a link between the amount of available land and its returns for the benefit of the GTM model, which uses returns as an exogenous input, while at the same time we use the GTM model to create the response of agricultural land quantity to returns in land returns for the benefit of the GTAP model, which accepts change in available agricultural land as an exogenous input. Specifically, we solve both models concurrently for variable qo for land in the GTAP model, measuring the change in available land, and the parameter RENTA in the GTM model, which defines the return to land, and which are internally consistent, i.e., the resulting rent change from agricultural land changes in the GTAP model are exactly the same as the changes in rents and the associated changes in agricultural land (calculated as the non-forested area from the total available land). Solving both models together in this way therefore allows us to expand their descriptive power.

We achieve a combined solution of GTAP and GTM by passing outputs back and forth between the models until a dual-state equilibrium is achieved. Establishing a connection between the GTAP model and the GTM model requires that we model the return on land using the GTAP model and feed those results into the GTM model as exogenous inputs. The GTM model then endogenously determines the amount of land available for crop and livestock production, and we feed that back into the GTAP model as an exogenous input. The land conversion considered in both models is fairly limited individually, but much more flexible in the combined system. While the standard GTAP model only allows the conversion of land among crop and pasture use (using a CET supply structure), the GTM model only considers the conversion between forest and agricultural land. Together, however, the two models can simulate the conversion of land across agricultural (crop and pasture) and forest use.

It is important to note that while the GTM model is a forward-looking, dynamic model, the standard GTAP model is a comparative static model. However, even though the two models operate with many different assumptions, the variables that we use to connect them—land quantity and land returns—are strictly exogenous to one or the other model. For this reason the connection between the models that we create does not interfere with any of their assumptions, since we are only changing the exogenous information that is not calculated by the models, i.e., the GTAP model modifies the exogenous rent values in the GTM model while the GTM model modifies the exogenous amount of the available agricultural land in the GTAP model.

On the software level, we establish the connection between the models using an R script, which is capable of handling other software, including generating required inputs, executing it and reading in any outputs. This is required to operate the GTAP model, which is written in GEMPACK, and the GTM model, which is written in GAMS. As GEMPACK and GAMS were developed long before software integration became important or even possible, we use much more modern technologies made available through the R software [30] to create the user functions that execute both models, collect their outputs, and automatically create any required input files.

## Changes under the baseline projections

To understand the impact of potential regional and global afforestation actions, an important first step was creating a realistic baseline scenario that depicts the likely changes over the decade under study, 2023–2033. Data for this were drawn from CEPII [28]. To build our scenario, we use two of their variables that we determined to be key: population and GDP (targeted by endogenous uniform productivity change). The CEPII projections do not contain any information on land use. To build a more complete baseline, we also include the projections on forested land change included in the GTM. Using the GTAP model in connection with the GTM model (to assure that the changes in land are consistent between the two models, which means that the change in agricultural land calculated by the GTM model will be exactly the same change in available agricultural land in the GTAP model), we calculate the overall change in factor quantity/productivity growth required to achieve the targeted values in 2033. In Table 1, we show the projected population and GDP growth per region. GDP increases for all regions of the world with the largest potential percentage changes occurring in South Asia (+ 308.6%), China (+ 189.8%), and Sub-Saharan Africa (+ 172.9%). Population also is projected to grow in most regions with the largest changes happening in Sub-Saharan Africa (+ 55.7%). Japan, Russia, and East Asia are projected to see their populations decline by 10.4%, 4.7%, and 2.6%, respectively. As for the projected change in agricultural land, our baseline projections show the largest increases in Oceania (+ 27.6%), Other Latin America (+ 25.8%), Sub-Saharan Africa (+ 17.4%), Central America (+ 13.9%), and Brazil (+ 13.9%). Japan is the only nation that sees a significant decline (− 34.9%) in agricultural land, which is likely tied in part to population trends.

**Table 1:** GDP, population, and agricultural land growth under the baseline scenario (Source: CEPII and GTM)

	Real GDP (%)	Population (%)	Agricultural land (%)
Oceania	52.7	24.0	27.6
China	189.8	0.8	0.0
Japan	20.6	− 10.4	− 34.9
East Asia	46.0	− 2.6	− 1.1
Southeast Asia	138.5	15.0	11.7
South Asia	308.6	17.8	12.8
Canada	41.3	15.2	2.6
United States	38.6	10.6	− 0.5
Central America	74.1	18.3	16.7
Brazil	20.8	7.8	13.9
Other Latin America	64.8	15.8	25.8
Western & Central Europe	25.4	0.9	4.5
Other Europe	113.1	8.7	3.1
Russia	41.8	− 4.7	4.4
Sub-Saharan Africa	172.9	55.7	17.4
North Africa/Middle East	58.2	27.8	7.8
ROW	44.4	36.8	0.0

Focusing on the absolute area of the forestland, as generated by the GTM model, we estimate that between 2023 and 2033, 144.2 million hectares (3.6%) of global forests are projected to be converted to agricultural lands with the largest decline expected to happen in Sub-Saharan Africa, which is estimated to lose 39.7 million hectares (7.0%) of its forestland (Table 2). Southeast Asia, Brazil, and Other Latin America also see forestland shrink, with decreases of 22.6 million hectares (9.3%), 19.0 million hectares (3.8%), and 23.5 million hectares (6.7%), respectively. Conversely, the United States is estimated to add 1.3 million hectares (0.5%) to its forestland.

**Table 2:** Forestland by region in 2023 and estimated change in 2033 relative to 2033 baseline scenario

	Forestland in 2023	Forestland in 2033	∆ (Mha)	∆ (%)
United States	248.4	249.7	1.3	0.5
China	164.6	164.8	0.1	0.1
Brazil	498.2	479.2	− 19.0	− 3.8
Canada	412.8	407.9	− 5.0	− 1.2
Russia	838.1	828.6	− 9.5	− 1.1
Western & Central Europe^d^	186.7	175.5	− 11.1	− 6.0
Other Europe^e^	40.1	36.1	− 4.1	− 10.1
South Asia	47.8	42.5	− 5.3	− 11.0
Central America	94.6	91.2	− 3.4	− 3.6
Other Latin America^c^	348.5	325.0	− 23.5	− 6.7
Sub-Saharan Africa	564.6	525.0	− 39.7	− 7.0
Southeast Asia^a^	243.9	221.3	− 22.6	− 9.3
Oceania^b^	199.2	196.1	− 3.1	− 1.5
Japan	23.7	26.9	3.2	13.7
North Africa/Middle East	33.8	30.9	− 2.9	− 8.5
East Asia	14.5	14.6	0.1	0.7
Total	3959.5	3815.3	− 144.2	− 3.6

^a^Southeast Asia includes Brunei Darussalam, Cambodia, Indonesia, Laos, Malaysia, Myanmar, Philippines, Singapore, Thailand, Timor-Leste, and Vietnam

^b^Oceania includes Australia, Cook Islands, Fiji, French Polynesia, Kiribati, Marshall Islands, Micronesia, Nauru, New Caledonia, New Zealand, Palau, Papua New Guinea, Samoa (Western Samoa), Solomon Islands, Tonga, Tuvalu, and Vanuatu

^c^Other Latin America includes Argentina, Bolivia, Czech Republic, Chile, Colombia, Ecuador, the Falkland Islands, French Guiana, Guyana, Paraguay, Peru, South Georgia, the South Sandwich Islands, Suriname, Uruguay, and Venezuela

^d^Western & Central Europe includes Austria, Belgium, Bulgaria, Croatia, Denmark, Estonia, Finland, France, Hungary, Iceland, Italy, Ireland, Germany, Greece, Latvia, Liechtenstein, Lithua- nia, Luxembourg, Malta, Monaco, Montenegro, Netherlands, North Macedonia, Norway, Poland, Portugal, Romania, Serbia, Slovakia, Slovenia, Spain, Sweden, Switzerland, Turkey, and the United Kingdom

^e^Other Europe includes Albania, Armenia, Azerbaijan, Belarus, Bosnia and Herzegovina, Georgia, Kosovo Kazakhstan, Kyrgyzstan, Moldova, Tajikistan, and Ukraine

## Modeling the impact of possible measures to mitigate deforestation/promote afforestation

Because the area of forestland is mostly determined by the relative returns of land in agriculture and forestry, in our policy scenario we modify the return to land in agriculture by applying a uniform global tax on the use of land in the production of agricultural output. This tax, imposed as a percentage of the rent, would create a wedge between the returns to landowners and the cost to the land users, when land is used in agricultural production (i.e., crops, pasture). Naturally, it is important to note that such a tax may face some implementation issues for landowners who also use the land in agricultural production, because it would require an assessment of the value of the land. In our scenario, the tax is used to measure the potential impact on deforestation in the period of 2023–2033.

Solving the two models together shows that it would require a tax of roughly 70 percent to be assessed on the use of land for the deforestation rate to fall globally to zero. The economic impact of such a policy measure would not be limited to the cost of the tax, it would also impact food production and its prices. For example, with this policy in place, most regions of the world would see significant declines in the amount of agricultural land in 2033 relative to the baseline scenario where land conversion was not taxed. For the United States, there would be 2.1 percent less agricultural land (Table 3). Larger decreases would happen in other major agricultural production regions such as China (− 2.5 percent), South Asia (− 7.9 percent), Brazil (− 8.6 percent), and Russia (− 9.3 percent).

**Table 3:** Changes in key variables in 2033 under a uniform land tax of 70% and relative to 2033 baseline scenario

	Real GDP (%)	Agr. land (%)	Agr. prices (%)	Agr. output (%)
Oceania	− 0.1	− 23.9	2.8	− 2.5
China	0.0	− 2.5	1.1	0.0
Japan	− 0.3	− 60.8	7.8	− 6.3
East Asia	− 0.2	− 15.0	5.4	− 5.0
Southeast Asia	− 0.3	− 9.0	2.9	− 1.7
South Asia	− 0.3	− 7.9	3.0	− 1.0
Canada	0.0	− 7.9	0.7	1.9
United States	0.0	− 2.1	0.7	1.0
Central America	− 0.2	− 15.6	2.7	− 2.0
Brazil	− 0.1	− 8.6	1.3	0.6
Other Latin America	− 0.4	− 23.1	3.8	− 3.2
Western & Central Europe	0.0	− 6.1	1.0	0.7
Other Europe	− 0.1	− 3.2	1.3	0.3
Russia	− 0.1	− 9.3	1.1	0.3
Sub-Saharan Africa	− 0.2	− 4.0	1.4	− 0.2
North Africa/Middle East	− 0.1	− 10.2	2.0	− 0.7
ROW	0.0	0.0	0.8	0.9

These reductions in the amount of agricultural land would then impact the quantity of agricultural goods that could be produced. For example, the 15.0 percent decrease in agricultural land in East Asia and the 9.0 percent decrease in agricultural land in Southeast Asia have the potential to reduce agricultural production by 5.0 percent and 1.7 percent, respectively. Surprisingly, Brazil and the United States, despite projected decreases in available agricultural land compared to the baseline 2033 scenario, are estimated to see increased agricultural output of 0.6 percent and 1.0 percent, respectively. This happens when the sharp output decline in other regions prompts increased intensity of production in the United States and Brazil.

**Fig. 1:**
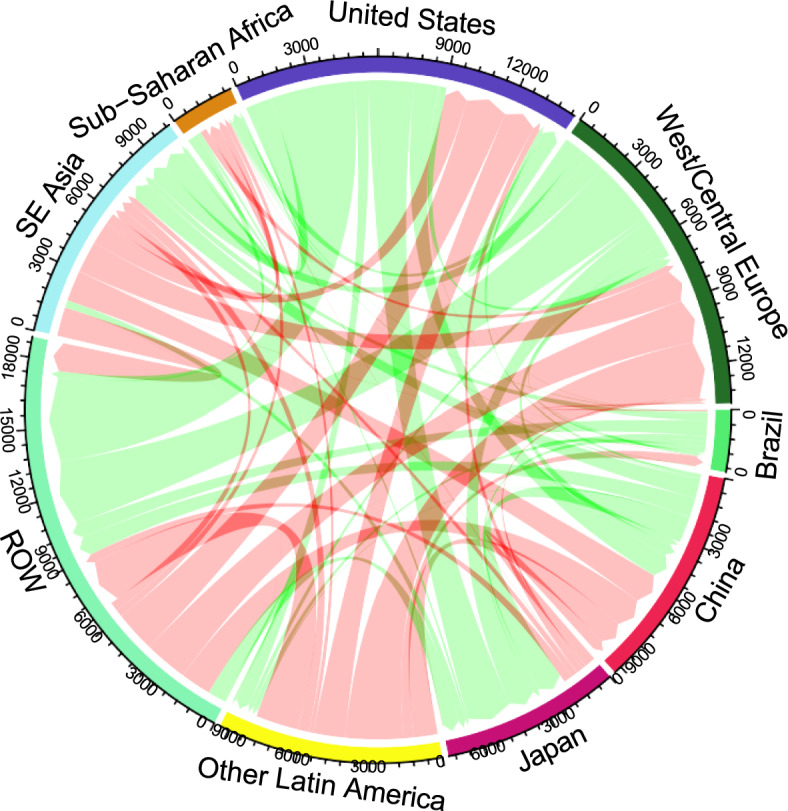
Changes in ag trade volumes (red arrows denote reductions, green arrows denote increases; for presentation purposes, the regions with smaller impacts are aggregated in ROW)

When we examine how the policy impacts exports of agricultural goods, we find that exports from Japan and the United States are estimated to grow the most, increasing 8.8 percent and 3.5 percent, respectively. The regions where agricultural exports  are projected to drop the most are East Asia, South Asia, Southeast Asia, and Central America, which see decreases of 14.5 percent, 5.9 percent, 5.1 percent, and 5.0 percent, respectively. Appendix Table A1 6 shows the details of the bilateral changes in agricultural trade; in Figure 1 we show some of the largest changes in the volumes of trade in a graphical form, showing the large (relative to the total trade) increases from the Unites States, Western and Central Europe, and the reductions out of Southeast Asia and Other Latin America. The change in the amount of forested land in each region with the mitigation policy in place is shown in (Table 4). Russia sees the largest increase in forestland (11.6 Mha), and Sub-Saharan Africa sees the largest drop (29.0 Mha).

**Table 4:** Percentage change in the amount of forested land in 2033 with deforestation mitigation policy in place relative to 2033 baseline scenario (Source: Authors’ calculations)

	Forestland in 2023	Forestland in 2033	∆ (Mha)	∆ (%)
United States	248.4	254.8	6.4	2.6
China	164.6	174.8	10.2	6.2
Brazil	498.2	492.2	− 6.0	− 1.2
Canada	412.8	423.2	10.4	2.5
Russia	838.1	849.7	11.6	1.4
Wesern & tCentral Europe	186.7	191.1	4.5	2.4
Other Europe	40.1	40.3	0.2	0.4
South Asia	47.8	46.2	− 1.6	− 3.3
Central America	94.6	94.9	0.3	0.3
Other Latin America	348.5	348.1	− 0.3	− 0.1
Sub-Saharan Africa	564.6	535.6	− 29.0	− 5.1
Southeast Asia	243.9	240.5	− 3.5	− 1.4
Oceania	199.2	199.0	− 0.1	− 0.1
Japan	23.7	23.5	− 0.1	− 0.6
North Africa/Middle East	33.8	34.9	1.1	3.4
East Asia	14.5	15.8	1.3	9.3
Total	3959.5	3964.9	5.3	0.1

Overall, the tax policy, which results in zero net deforestation globally, has a projected negative economic impact on all regions except Canada, which sees an increase in consumer well-being of $0.4 billion, respectively (Table 5). Consumer well-being is a measurement of equivalent variation and here measures the benefit/harm consumers experience in terms of income as a result of the policy changes. The United States is estimated to see well-being drop by more about $1 billion. The most negatively impacted regions are South Asia (− $27.8 billion), China (− $15.9 billion), and Southeast Asia (− $13.9 billion). In total, this tax-driven policy is estimated to decrease global consumer well-being in 2033 by $119.7 billion relative to the scenario where no tax on forestland conversion was in place. It is important to note that this reduction in the consumer welfare only stems from the market changes, i.e., prices and incomes, and it excludes any additional benefits that could come into being through afforestation.

**Table 5:** Change in consumer well-being in 2033, by region, in billions of U.S. dollars, with deforestation mitigation policy in place relative to 2033 baseline scenario

	Baseline (B$)	Scenario (B$)	∆ (B$)
Oceania	863.7	861.8	− 1.9
China	23,275.9	23,260.0	− 15.9
Japan	1052.6	1036.1	− 16.5
East Asia	1168.4	1163.0	− 5.4
Southeast Asia	3886.2	3872.4	− 13.9
South Asia	9493.4	9465.6	− 27.8
Canada	713.1	713.5	0.4
United States	7497.2	7496.4	− 0.9
Central America	1241.2	1236.4	− 4.8
Brazil	423.0	422.4	− 0.6
Other Latin America	1242.6	1233.2	− 9.4
Western & Central Europe	5126.7	5118.8	− 8.0
Other Europe	839.3	838.7	− 0.6
Russia	706.2	704.9	− 1.3
Sub-Saharan Africa	2766.7	2758.8	− 7.8
North Africa/Middle East	1715.9	1710.7	− 5.3
ROW	13.9	13.9	0.0
Total	62,026.3	61,906.6	− 119.7

Much of this loss in well-being is the result of the projected decline in the availability of land for production. Other losses come from the tax which create a deadweight loss for the world economy.

## Discussion

This analysis shows that, at an estimated tax rate of 70.3 percent of the value of each hectare of land that is converted to agricultural land, global net deforestation is estimated to drop to roughly zero by 2033, preserving 144.2 million hectares of forestland that otherwise would have been converted to agricultural land. In the United States, as USDA-NASS [Land Values 2022 Summary (August 2022)] valued the average hectare of farmland in 2022 at $1537.8, the tax would equate to $1081.1/hectare. In Brazil, CEIC valued the average price of Brazilian land at roughly 2500 Brazilian Reals, which would put the tax at roughly $82.2 per hectare. The economic price of this would be transferred to consumers through lower agricultural output and higher market prices. Overall, this land tax is estimated to reduce consumer well-being by $119.7 billion or $13.78 per person in 2033.

We also show the utility of joining two major economic models. GTAP has long been valued for its ability to provide robust information on global trade flows and prices, and GTM has proven its utility in understanding the impact of forestry decisions on the carbon sink and land rents. By combining these models through the use of R and allowing the models to pass inputs and outputs back and forth iteratively, the benefits of both models have been maintained and their weaknesses greatly minimized. This advance holds great promise for advancement on a variety of fronts for researchers and policymakers interested in climate change, agricultural trade, and their inter-dependencies.

## Conclusions

According to CEPII’s projections, the growth of the global economy in the next ten years is expected to result in tremendous increases in global and regional GDP accompanied by a moderate growth in population. Another set of projections by GTM suggests that this growth would also mean significant reductions in the world’s forests because they would be converted to agricultural land. The model predicts a loss of over 144 million hectares of forest before 2033.

By combining two well-known global models—the GTAP model that describes the world markets and trade, and the GTM model that describes the impact of economic and population growth along with land rents on the amounts of forested areas—we were able to estimate the size of the tax on agricultural land needed to prevent deforestation in the coming ten years through reducing the returns to land used in agriculture. We find that even though the tax is substantial at about 70 percent, it results in only very small reductions in the projected growth in global GDP and welfare. We also observe that the reduction in available agricultural land results in reduced agricultural output and higher prices, which might require additional policy actions to avoid negative impacts in terms of food security.

We note that a uniform global tax reducing the returns to land to the point of preventing global deforestation would produce highly differentiated impacts around the world’s regions. Some regions, such as China, Russia, Canada and the United States would increase their forested areas as a result of the tax, while the regions with the greatest deforestation pressures, such as Sub-Saharan Africa, Brazil and Southeast Asia would only reduce the rate of their deforestation.

We hope that the framework that we developed in this paper can be used for the analysis of any other policy scenarios that wish to assess the impact of reducing deforestation with standard policy tools.

## Data Availability

No datasets were generated or analysed during the current study.

## Appendix

See Table 6

**Table 6:** Change in trade, millions of USD (2023 prices). Exporters in rows, importers in columns

	Oceania	China	Japan	East Asia	SE Asia	South Asia	Canada	United States	Central America	Brazil	Other Latin America	West/Central Europe	EU II	Russia	Sub-Saharan Africa	North Africa/Middle East	ROW
Oceania	− 152	− 958	223	− 13	− 135	− 46	− 63	− 378	− 30	− 2	− 2	− 490	− 22	− 14	− 53	− 211	− 14
China	74	148	1163	488	855	131	4	145	34	26	52	20	6	33	59	81	1
Japan	− 48	− 512	0	− 262	− 268	− 5	− 32	− 288	− 6	− 4	− 5	− 161	− 3	− 9	− 34	− 42	0
East Asia	− 67	− 559	− 216	− 63	− 353	− 13	− 40	− 252	− 19	− 5	− 10	− 153	− 9	− 32	− 28	− 131	− 1
SE Asia	− 171	− 1364	326	− 53	− 716	− 284	− 139	− 996	− 55	− 31	− 9	− 1426	− 57	− 106	− 509	− 411	− 19
South Asia	− 18	− 132	27	− 9	− 201	− 125	− 52	− 381	− 22	− 4	− 4	− 602	− 67	− 67	− 246	− 576	− 20
Canada	17	279	536	113	153	164	0	927	95	6	121	65	2	3	39	80	0
United States	159	1075	2017	1148	1058	339	465	40	1330	41	615	339	16	10	114	309	9
Central America	− 6	− 53	62	1	− 9	− 1	− 178	− 1746	− 260	− 3	4	− 558	− 8	− 8	− 31	− 32	0
Brazil	7	263	253	165	323	208	− 6	25	27	0	220	− 80	0	13	47	271	1
Other Latin America	− 70	− 1067	− 28	− 93	− 493	− 216	− 218	− 1436	− 293	− 445	− 542	− 2551	− 39	− 342	− 206	− 626	− 19
West/Central Europe	296	499	1180	574	783	172	58	796	167	117	223	4117	134	207	439	1262	16
EU II	1	20	17	27	61	215	0	2	2	0	1	− 44	14	45	13	127	1
Russia	1	42	54	86	48	58	0	10	5	0	7	7	50	0	37	136	5
Sub-Saharan Africa	10	10	133	43	207	177	− 10	4	4	7	7	− 299	− 2	2	84	69	− 1
North Africa/Middle East	2	− 14	18	7	18	79	− 9	− 23	− 1	0	2	− 184	− 38	− 19	− 49	− 148	− 10
ROW	0	2	2	1	7	1	0	1	0	0	0	17	3	4	0	13	0

## Footnotes

1 Downloaded from https://u.osu.edu/forest/gtm/.

2 https://www.gtap.agecon.purdue.edu/models/current.asp.
